# Supplementary material for: Human Adenovirus and Influenza A Virus Exacerbate SARS-CoV-2 Infection in Animal Models
Source: Microorganisms. 2023 Jan 11;11(1):180. doi: 10.3390/microorganisms11010180 (PMC9860643; doi:10.3390/microorganisms11010180)
Supplement: Supplementary file 1 [file microorganisms-11-00180-s001.zip › Svyat_Figure S1.pdf]

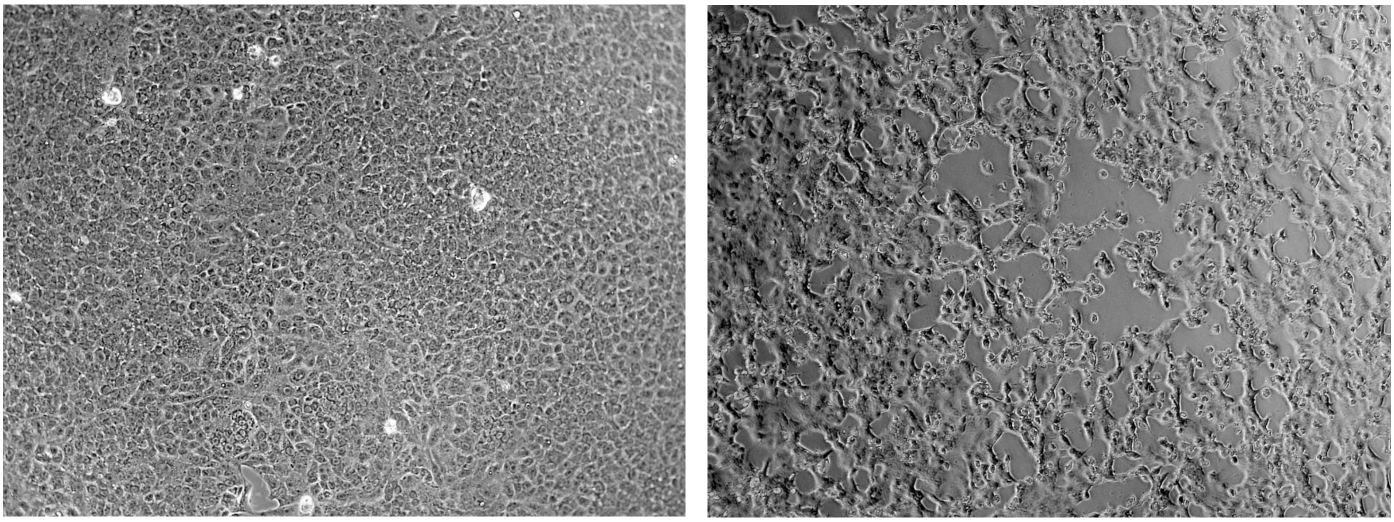

a) b)

Figure S1: SARS-CoV-2 CPE in the Vero E6 cell monolayer.

a) Mock-infected cells; b) SARS-CoV-2 infected cells (MOI 0.1 $TCID_{50}$, 48 h post infection). Magnification × 100.
