# Supplementary material for: Human Adenovirus and Influenza A Virus Exacerbate SARS-CoV-2 Infection in Animal Models
Source: Microorganisms. 2023 Jan 11;11(1):180. doi: 10.3390/microorganisms11010180 (PMC9860643; doi:10.3390/microorganisms11010180)
Supplement: Supplementary file 1 [file microorganisms-11-00180-s001.zip › Svyat_Table S1.pdf]

Table S1: Primers used in this study.

Target	Forward	Reverse
SARS-CoV-2 1ab	TAGACATCATGCTAATGAGTACAGAT	TGAAGTCTTGTAAAAGTGTTCCAG
	Probe ROX-GCTTATAACATGATGATCTCAGCTGGC-BHQ1	
HAdV-5 hexon	CCCITCGATGMTGCCCC	CIACGGGIACIAARCGCA
	Probe ROX-CCTGGCCCGAGATGTGCAT-BHQ1	
IAV M	The IAV primers and probe sequences are published as part of the AmpliSens® Influenza virus A/B-FL detection kit (Moscow, Russia, Reg.# RU #FSR 2009/05010).	
