# Supplementary material for: Human Adenovirus and Influenza A Virus Exacerbate SARS-CoV-2 Infection in Animal Models
Source: Microorganisms. 2023 Jan 11;11(1):180. doi: 10.3390/microorganisms11010180 (PMC9860643; doi:10.3390/microorganisms11010180)
Supplement: Supplementary file 1 [file microorganisms-11-00180-s001.zip › Svyat_Table S2.pdf]

Table S2: Determination of the SARS-CoV-2 replicative activity in the lungs of monoinfected and coinfected with HAdV-5 hamsters by the formazan-based MTT assay.

Dilutions of lung homogenates	SARS-CoV-2	SARS-CoV-2/HAdV-5	HAdV-5/3 days/SARS-CoV-2	Mock-infected
		Optical Density (OD_{570})		
10^{0}	0.123±0.024	0.137±0.029	0.144±0.035	1.230±0.215
10^{-1}	0.143±0.035	0.153±0.044	0.147±0.041	1.244±0.207
10^{-2}	0.135±0.041	0.129±0.037	0.157±0.045	1.233±0.217
10^{-3}	0.128±0.037	0.125±0.041	0.121±0.029	1.250±0.220
10^{-4}	0.155±0.046	0.167±0.044	0.141±0.035	1.227±0.195
10^{-5}	0.513±0.094	0.453±0.087	0.537±0.115	1.242±0.211
10^{-6}	0.836±0.144	0.723±0.127	0.870±0.163	1.239±0.223
10^{-7}	1.248±0.234	1.237±0.222	1.229±0.192	1.243±0.227
		Virus Titer (lg $TCID_{50}$/g)		
	6.43±0.36	6.60±0.44	6.27±0.32	<1.00

Data are the mean ±SEM of 2 independent MTT tests (3 replicates per point). lg$TCID_{50}$ values were determined according to the method of Kärber as modified [1].

1. Ashmarin I.P., Vorob'ev A.A. *Statistical Methods in the Microbiological Research.* State Press of Medical Literature; Leningrad, USSR: 1962. pp. 85–104.
