# Supplementary material for: Human Adenovirus and Influenza A Virus Exacerbate SARS-CoV-2 Infection in Animal Models
Source: Microorganisms. 2023 Jan 11;11(1):180. doi: 10.3390/microorganisms11010180 (PMC9860643; doi:10.3390/microorganisms11010180)
Supplement: Supplementary file 1 [file microorganisms-11-00180-s001.zip › Svyat_Table S3.pdf]

Table S3: Determination of the HAdV-5 replicative activity in the lungs of monoinfected and coinfected with SARS-CoV-2 hamsters by the formazan-based MTT assay.

Dilutions of lung homogenates	SARS-CoV-2	SARS-CoV-2/HAdV-5	HAdV-5/3 days/SARS-CoV-2	Mock-infected
		Optical Density (OD_{570})		
10^{0}	0.118±0.023	0.120±0.020	0.123±0.025	1.355±0.229
10^{-1}	0.123±0.034	0.132±0.031	0.135±0.039	1.349±0.237
10^{-2}	0.137±0.036	0.127±0.026	0.147±0.040	1.340±0.234
10^{-3}	0.145±0.049	0.136±0.032	0.479±0.079	1.350±0.243
10^{-4}	0.133±0.037	0.585±0.094	1.121±0.221	1.290±0.215
10^{-5}	0.143±0.040	0.913±0.174	1.255±0.235	1.322±0.227
10^{-6}	0.515±0.133	1.325±0.243	1.270±0.263	1.269±0.243
10^{-7}	1.115±0.223	1.348±0.234	1.229±0.242	1.310±0.259
		Virus Titer (lg $TCID_{50}$/g)		
	6.77±0.45	4.86±0.37	3.44±0.30	<1.00

Data are the mean ±SEM of 2 independent MTT tests (3 replicates per point). lg$TCID_{50}$ values were determined according to the method of Kärber as modified [1].

1. Ashmarin I.P., Vorob’ev A.A. *Statistical Methods in the Microbiological Research.* State Press of Medical Literature; Leningrad, USSR: 1962. pp. 85–104.
