# Supplementary material for: Human Adenovirus and Influenza A Virus Exacerbate SARS-CoV-2 Infection in Animal Models
Source: Microorganisms. 2023 Jan 11;11(1):180. doi: 10.3390/microorganisms11010180 (PMC9860643; doi:10.3390/microorganisms11010180)
Supplement: Supplementary file 1 [file microorganisms-11-00180-s001.zip › Svyat_Table S4.pdf]

Table S4: The evaluation of lung pathology for infected animals

	Infection by:																			
**The pathological lesions**	**Control**		**SARS-CoV-2**			**HAdV-5**			**IAV**			**SARS-CoV-2/HAdV-5**			**HAdV-5/3 day/SARS-CoV-2**			**IAV/ SARS-CoV-2**		
Interstitial pneumonia involving neutrophils and heterophiles	0	0	2	2	2	1	2	1	1	2	2	2	3	2	2	3	3	3	2	3
Acute diffuse alveolar damage, necrosis of alveolar epithelial cells	0	0	1	2	2	0	0	0	1	0	1	2	2	2	2	3	2	2	2	2
Bronchitis with necrosis of bronchial epithelial cells	0	0	2	1	2	0	1	0	1	1	1	2	1	2	1	1	2	1	2	2
Hyperplasia of the bronchial epithelium	0	0	1	1	1	0	0	0	0	1	0	1	1	1	1	1	1	1	1	1
Endothelitis with necrosis and desquamation of endothelial cells	0	0	1	1	1	0	0	0	0	1	0	1	1	1	1	1	2	1	2	1
Perivascular edema and perivascular lymphocytic infiltration	0	0	1	0	2	1	1	0	1	1	1	2	3	2	2	2	2	2	2	2
Intraalveolar hemorrhages	0	0	1	1	1	0	0	1	1	1	0	1	1	1	1	1	2	2	2	2

Note. The pathological changes of the lung on a scale: 0 - absence of pathology; 1- mild lesions; 2 - moderately lesions; 3 – pronounced lesions [1]. Mock-infected (2 hamsters), SARS-CoV-2, HAdV-5, IAV, SARS-CoV-2/HAdV-5, HAdV-5/3 day/ SARS-CoV-2, IAV/SARS-CoV-2 (3 hamsters). Three evenly distributed sections from each lung were scored for various parameters.

1. Gruber AD, Osterrieder N, Bertzbach LD, Vladimirova D, Greuel S, Ihlow J, Horst D, Trimpert J, Dietert K. Standardization of Reporting Criteria for Lung Pathology in SARS-CoV-2-infected Hamsters: What Matters? Am J Respir Cell Mol Biol. 2020 Dec;63(6):856-859. doi: 10.1165/rcmb.2020-0280LE. PMID: 32897757
